# Gradient Microstructure and Texture Formation in a Metastable Austenitic Stainless Steel during Cold Rotary Swaging

**DOI:** 10.3390/ma16041706

**Published:** 2023-02-17

**Authors:** Dmitrii Panov, Egor Kudryavtsev, Stanislav Naumov, Denis Klimenko, Ruslan Chernichenko, Vladimir Mirontsov, Nikita Stepanov, Sergey Zherebtsov, Gennady Salishchev, Alexey Pertcev

**Affiliations:** 1 Laboratory of Bulk Nanostructured Materials, Belgorod State University, 85 Pobeda St., 308015 Belgorod, Russia; 2 Department Chief Metallurgist, Perm Scientific-Research Technological Institute, 41 Geroev Khasana St., 614990 Perm, Russia

**Keywords:** metastable austenitic stainless steel, gradient microstructure, texture, strain-induced martensitic transformation, microhardness distribution, finite element simulation, rotary swaging

## Abstract

The paper aimed to study the evolution of the microstructure and texture gradient of a 321-type metastable austenitic stainless steel during cold rotary swaging. Cold rotary swaging was carried out with a reduction of up to 90% at ambient temperature. Pronounced gradients of the α’-martensite volume fraction, the axial texture of austenite (〈111〉 and 〈001〉) and α’-martensite (〈101〉), and non-uniform microhardness distribution along the rod diameter were obtained after a reduction of 80–90%. According to the finite element analysis, moderate tensile stresses were attained in the center, whereas high compressive stresses operated at the edge. Due to water cooling of the rod surface and heating of the rod center during processing, a temperature gradient was also derived. Features of strain-induced martensitic transformation, microstructure and texture evolution, and non-uniform hardening during cold rotary swaging were discussed.

## 1. Introduction

Metastable austenitic stainless steels (MASSs) possess attractive corrosion resistance, excellent ductility, and good impact toughness [1,2,3]. However, low yield strength is expected. To increase the yield strength value, various conventional deformation techniques have been applied [4,5,6,7,8]. However, as has been shown earlier [8,9], the strengthening of MASS by plastic deformation results in decreasing the ductility and impact toughness. The strength–ductility trade-off might be overcome via novel microstructural design by producing gradient structures [10]. Generally, the gradient structure consists of layers with a gradual change in the grain size, phase volume composition, or phase morphology from the surface to the workpiece core. In such structures, soft/hard interfaces can be distinguished that additionally lead to the multiplication and accumulation of geometrically necessary dislocations and the development of back-stress hardening [11,12]. Furthermore, different grain sizes of metastable austenite are associated with various stability, which enhances the strain-hardening ability throughout multi-stage martensitic transformation [13,14]. 

Nowadays, torsional deformation [15,16,17], surface mechanical rolling treatment (SMRT) [18,19,20], ultrasonic impact treatment [21,22], and surface mechanical attrition treatment [13,23] have been applied to produce the gradient structure. For instance, a good strength–ductility combination of an AISI 304 MASS can be received by obtaining austenitic domains with various dimensions in depth [16]. For an AISI 316L MASS with the gradient structure, the inhibition of crack nucleation and the accommodation of cyclic plastic strain amplitude were attained, which essentially enhance the low and high fatigue properties [18]. Aside from the excellent strength–ductility combination, SMRT and following annealing of the AISI 316L MASS resulted in increased corrosion resistance [19]. Evidently, due to the small sample dimensions, substantial laboratory applications may be considered for the above-mentioned techniques. 

On the other hand, rotary swaging may be considered as a promising industrial method with high performance for producing gradient materials via bulk-dominated plastic deformation [24]. For instance, gradients of axial two-component (〈001〉 and 〈111〉) austenitic texture and microstructure were detected in a 316-type austenitic stainless steel after cold rotary swaging [25]. Furthermore, cold rotary swaging of an AISI 321 MASS with a 90% reduction was accompanied by the formation of the α’-martensite volume fraction gradient from the rod core to the surface that, after low-temperature annealing, resulted in an extraordinary strength–toughness combination [26]. It is worth noting that close to hydrostatic compression, the capacity for the accumulation of high plastic strain without failure and the non-uniform stress condition and accumulation of plastic strain are the main features of swaging, affecting the structure and texture evolution [24,25,26,27]. Meanwhile, the temperature gradient is also expected to affect the texture development [28], strain-induced martensitic transformation, and twinning during processing [25,29]. Although many profound studies of the microstructure evolution during cold rotary swaging [30,31,32] have been conducted, the evolution of the microstructure and texture gradient of MASS during rotary swaging has not been investigated properly. Thus, the purpose of the current paper is to study the evolution of the microstructure and texture gradient of a MASS during cold rotary swaging.

## 2. Materials and Methods

### 2.1. Program Material Processing

**Figure 1:**
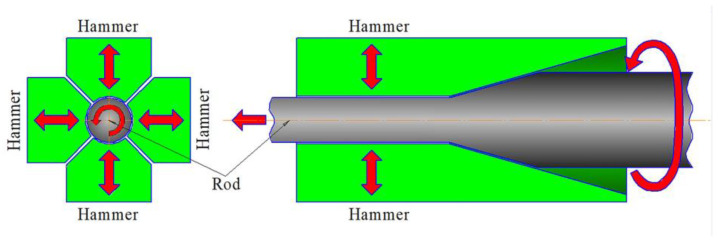
Scheme of cold rotary swaging. Red arrows indicate the movement direction of the rod and hammers during processing.

The industrial 321-type MASS was studied as the program material. The program material possessed the following chemical composition (wt.%): C–0.07%, Cr–18.75%, Ni–9.2%, Mn–1.12%, Si–0.39%, Ti–0.59%, S–0.019%, P–0.005%, Fe–balance. A rod with a diameter of 33 mm was received by hot rolling at 900–1220 °C with the following air-cooling to room temperature. Then, the rod was subjected to annealing at 1050 °C for 2 h with water cooling, which was considered an the as-received condition. After quenching, cold rotary swaging was carried out using an SXP-16 rotary swaging machine (GFM, Steyr, Austria) with a workpiece feeding rate of 180 mm/min, a stroke frequency of 1000 blows per minute, and a workpiece rotation of 25 rpm (rotations per minute) [25]. The scheme of rotary swaging is presented in Figure 1. The rod was water-cooled during processing. Five steps of swaging were performed: from ø33 to ø29 mm, from ø29 to ø25 mm, from ø25 to ø20 mm, from ø20 to ø14 mm, and from ø14 to ø11.5 mm, which equated to an ~20%, ~40%, ~60%, ~80%, and ~90% reduction in the cross-section area, respectively.

### 2.2. Structure and Texture Characterization

The cross-section microstructure was characterized using scanning electron microscopy (SEM) and transmission electron microscopy (TEM). For this purpose, samples were cut by an electrical discharge machine and prepared by means of conventional metallographic techniques, followed by electro-polishing in an electrolyte consisting of 5% perchloric acid, 35% butanol, and 60% methanol at room temperature with an applied voltage of 26 V. SEM observations were conducted using an FEI Nova NanoSEM 450 scanning electron microscope (FEI Company, Hillsboro, OR, USA) equipped with an EDAX Hikari electron backscatter diffraction (EBSD) camera (EDAX, Mahwah, NJ, USA). The EBSD analysis was carried out with a scanning step of 100 nm. To improve the EBSD data quality, only results with a confidence index (CI) ≥ 0.1 were used for the subsequent evaluation of texture. TEM was conducted using a JEOL JEM-2100 electron microscope (JEOL, Akishima, Tokyo, Japan) with an accelerating voltage of 200 kV. The structural parameters were estimated on bright- and dark-field TEM photographs. 

To evaluate the phase composition, X-ray diffraction (XRD) and eddy-current testing were applied. XRD was performed using a Rigaku Ultima-IV X-ray diffractometer (Rigaku, Akishima, Tokyo, Japan) in CuK_α_-radiation. Standard Bragg–Brentano geometry in the 2Θ angle ranging from 40 to 100° was applied. Estimation of the ferromagnetic BCC-phase volume fraction was carried out along the diameter using a FERRITSCOPE FMP30 eddy-current tester (Helmut Fischer Holding GmbH, Sindelfingen-Maichingen, Germany). First, the eddy-current tester was calibrated by the standard samples. Five or more measurements were conducted and averaged.

### 2.3. Microhardness Testing

The Vickers microhardness tests were carried out using a Wolpert 402MVD semi-automatic hardness tester (Wolpert, Maastricht, Netherlands) by a diamond pyramid indenter with a base angle of 136°. The testing was performed along the rod diameter of the cross-sections with a step of 0.5–0.7 mm, a load of 200 g, and a soaking of 15 s. The measurements were conducted in two perpendicular pathways along a rod diameter for each condition followed by the averaging of the results.

### 2.4. Finite Element Simulation

The finite element simulation was performed by the QFORM software (V. 9.0.7, QuantorForm, Moscow, Russia) according to the above-mentioned parameters of cold rotary swaging. Four hammers realizing deformation and a pusher performing the axial and rotational movement of the workpiece were included in the basic model. An adaptive finite element net with a mesh size of 0.2–6 mm was applied. The number of meshes was altered from 20,000 to 200,000. By reducing the heating of the workpiece, swaging was realized in a water environment with a temperature of 40 °C. The contact surface was used for the calculation of heat transfer between hammers and the workpiece with a heat transfer coefficient of 2500 W/m^2^K. Levanov’s law [33] was applied for the friction between the rod and hammer with a Levanov coefficient of 1.25 and a friction factor of 0.8.

## 3. Results

### 3.1. As-Received Condition

**Figure 2:**
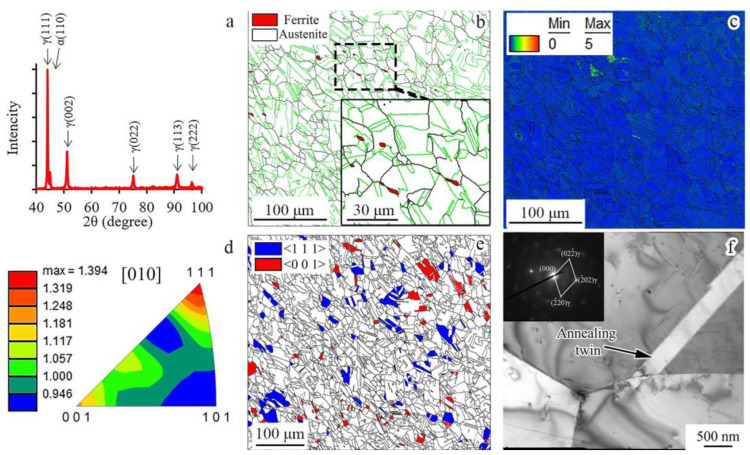
(**a**) XRD pattern, (**b**) phase map, (**c**) KAM map, (**d**) inverse pole figure, (**e**) crystal direction map, and (**f**) TEM photograph of the as-received material. In (**b**,**c**,**e**), the high-angles boundaries (misorientation more than 15°) are marked in black. Twin boundaries are marked in green in (**b**).

According to the XRD pattern (Figure 2a) and phase map (Figure 2b), face-centered cubic (FCC) and body-centered cubic (BCC) phases were detected in the as-received condition. Thin δ-ferrite (BCC-phase) grains were located along the austenite grains (Figure 2b). Austinite (FCC-phase) possessed equiaxed grains with an average size of ~10 μm. The fraction of the Σ3-type boundaries was 61% of high-angle boundaries (HAB), which might be referred to as an annealed condition. Kernel average misorientation (KAM) analysis of the as-received material presented the low uniform KAM level of the condition (Figure 2c). Meanwhile, a weak axial two-component (〈001〉 and 〈111〉) texture of austenite was detected (Figure 2d). On the inverse pole figure (IPF) (Figure 2d), the intensity of the 〈001〉 and 〈111〉 components was 1.2–1.3 MRD (multiple of random distribution). The volume fraction of the 〈001〉- and 〈111〉-oriented grains reached 4% and 6.5%, respectively (Figure 2e). According to the TEM observations, single dislocations and annealing twins within the austenitic grains were observed (Figure 2f).

### 3.2. Phase Composition Analysis

**Figure 3:**
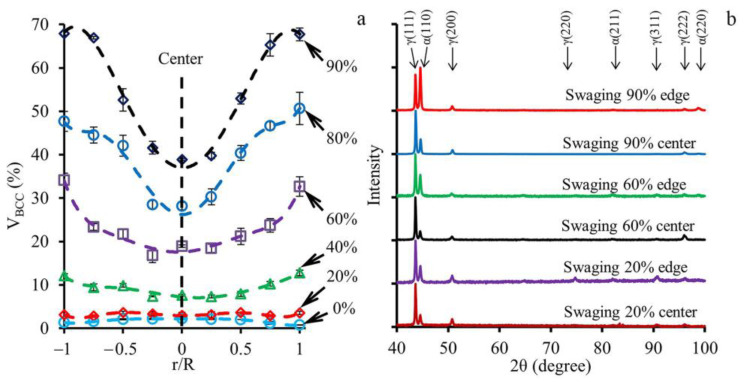
(**a**) Distribution of the ferromagnetic BCC-phase volume fraction (V_BCC_) along the diameter and (**b**) the XRD patterns after different swaging modes.

The distribution of the ferromagnetic BCC-phase along the rod diameter and the XRD patterns of the rod center and edge after different swaging modes are presented in Figure 3. In the as-received condition, the volume fraction of the BCC-phase was uniform along the diameter at ~2% (Figure 3a). After a reduction of 20% and 40%, the total BCC phase volume fraction was also uniform at 3% and 9%, respectively, which was associated with the development of strain-induced martensitic transformation (SIMT). The subsequent swaging was accompanied by the enhancement of SIMT and increasing the BCC-phase volume fraction. Therefore, after a 60% reduction, the pronounced gradient of the ferromagnetic BCC-phase volume fraction along the diameter was obtained (Figure 3a). In this instance, the BCC-phase volume fraction varied from ~18% in the center to ~33% at the edge. With a following increase in a reduction to 90%, the BCC-phase gradient became more pronounced where the BCC-phase volume fraction varied from ~40% in the center to ~70% at the edge. The XRD patterns were in good accordance with the results of the eddy-current tests (Figure 3b). Therefore, with an increase in reduction, the intensity of the (110)α peak increased, while the intensity of the (111)γ and (200)γ reflections decreased. Specifically, the (220)γ and (311)γ peaks completely disappeared after a reduction of 90%. It is worth noting that the intensity of the (110)α peak was more pronounced at the edge compared to the center (Figure 3b) due to a significant increase in the BCC-phase volume fraction to ~70% herein (Figure 3a).

### 3.3. EBSD Analysis 

**Figure 4:**
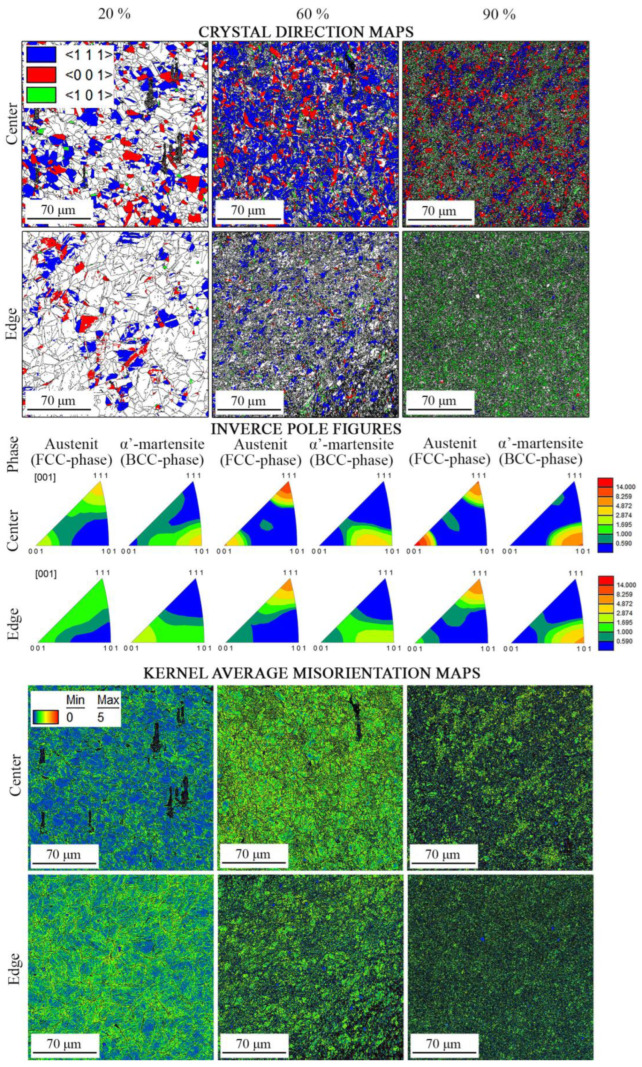
Crystal direction maps, IPFs, and KAM maps of the program material after cold rotary swaging with a reduction of 20%, 60%, and 90%, respectively. High-angle boundaries (misorientation more than 15°) in crystal direction and KAM maps are marked in black.

**Figure 5:**
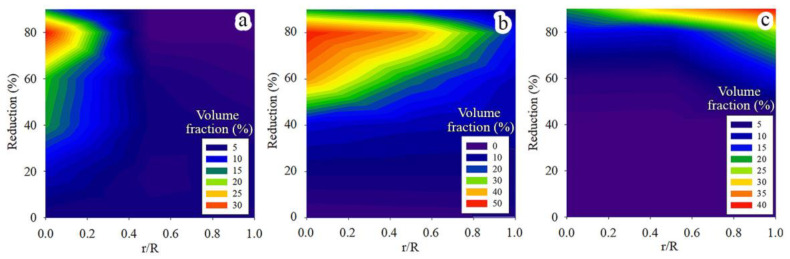
Volume fraction (%) of (**a**) $100_{FCC}$-, (**b**) $111_{FCC}$-, and (**c**) $110_{BCC}$-oriented grains against the reduction and radial distance (r/R)—texture maps.

Crystal direction maps, IPFs, and Kernel average misorientation maps of the program material after applying cold rotary swaging modes are shown in Figure 4. Texture maps were also established using EBSD data (Figure 5). The following trends of texture evolution during cold rotary swaging could be distinguished using the obtained results: (i) a strong two-component (〈111〉 and 〈001〉) axial texture of austenite was attained in the center that transferred into the one-component (〈111〉) texture to the edge (Figure 4); (ii) the volume fraction of the 〈111〉-oriented austenitic grains in the center increased to a maximum of ~50% after a 60% reduction with a subsequent decrease, while the volume fraction of 〈001〉-oriented austenitic grains grew to a maximum of 32% after a 80% reduction herein (Figure 5a,b); (iii) after a reduction of 60% and more, the volume fraction of 〈101〉-oriented α’-martensitic grains increased along the radial direction from the center to the edge (Figure 5c). The pole figures are also presented in Supplementary Materials Figures S1 and S2 that confirm the formation of a strong axial texture. Thus, the strong texture gradient of austenite and α’-martensite was attained after a reduction of 80–90%. It should be noted that a deviation between the results of texture analysis at the same point did not exceed 10%. Furthermore, Kernel average misorientation maps presented increased local lattice distortions in the center along grain boundaries after a reduction of 20%, while, at the edge, KAM increased along the deformation bands, grain boundaries, and mechanical twins (Figure 4). With a further reduction to 60% and more, high local lattice distortions were found throughout all KAM maps.

### 3.4. Microhardness Distribution

**Figure 6:**
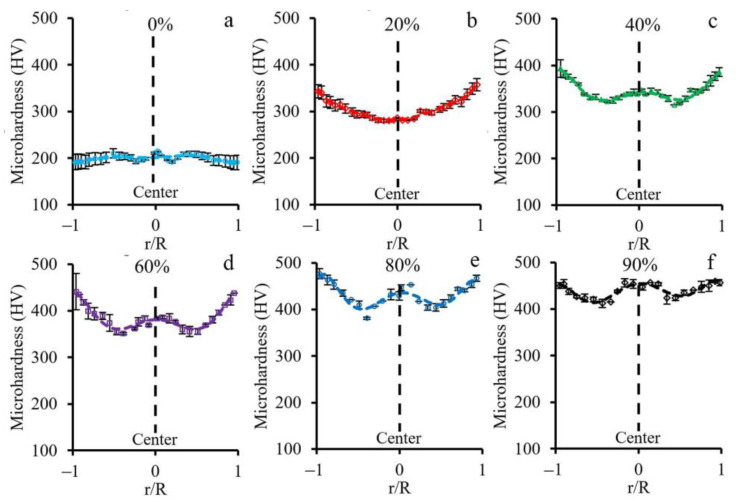
Microhardness distribution after cold rotary swaging with a reduction of (**a**) 0%, (**b**) 20%, (**c**) 40%, (**d**) 60%, (**e**) 80%, and (**f**) 90%.

The effect of the cold rotary swaging mode on the microhardness distribution is shown in Figure 6. The uniform distribution of microhardness at 190–200 HV was observed in the as-received material (Figure 6a). However, after a reduction of 20%, the microhardness of the center reached a level of ~280 HV, while the rod edge hardened to ~350 HV (Figure 6b). Thereby, the gradient microhardness distribution from the center to the edge was attained. On one hand, the following swaging with a reduction to 90% resulted in an increase in the microhardness throughout the rod cross-section (Figure 6c–f). On the other hand, the local maximum and minimum of microhardness were derived in the rod center and half radial distance, respectively. Sufficient increasing microhardness to the rod edge was also obtained.

### 3.5. TEM Observations

**Figure 7:**
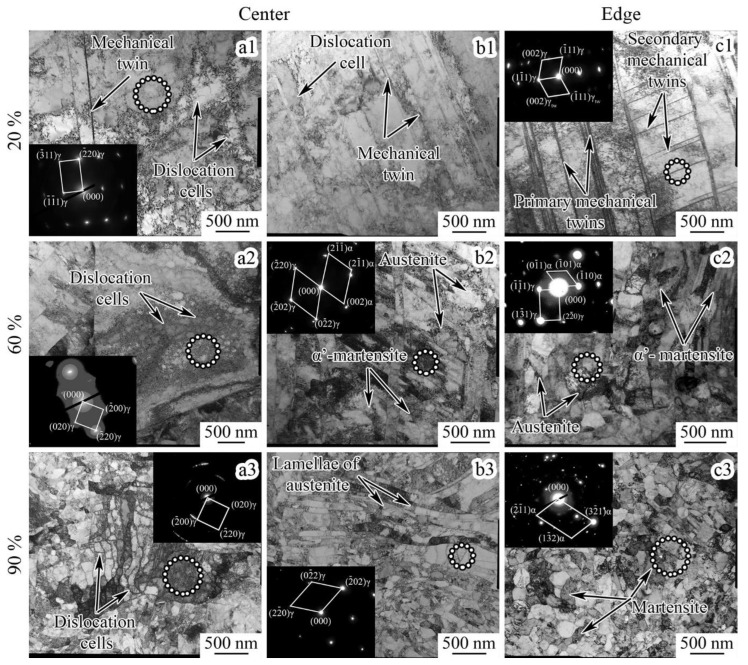
The TEM photographs and microdiffractions of the (**a1**–**a3**, **b1**–**b3**) rod center and (**c1**–**c3**) edge after cold rotary swaging with a reduction of (**a1**,**b1**,**c1**) 20%, (**a2**,**b2**,**c2**) 60%, and (**a3**,**b3**,**c3**) 90%. All insertions are microdiffraction patterns. The microdiffraction patterns were obtained from the areas indicated by dotted circles.

The results of the TEM observations are shown in Figure 7. After cold rotary swaging with a reduction of 20–40%, two types of austenite areas in the center might be distinguished: (i) grains with the dislocation cell microstructure and a few single mechanical twins inside (Figure 7a1); and (ii) grains with many mechanical twins of one twinning system and slightly pronounced dislocation cells (Figure 7b1). Meanwhile, at the edge, twinning occurred over several systems in most grains, and thereby a lamellar twin-matrix microstructure was obtained (Figure 7c1). Swaging with a reduction of 60% caused the formation of the lamellar austenite-martensitic microstructure in the center (Figure 7b2), while some areas still possessed the developed dislocation cell structure (Figure 7a2). However, the fragmentation of the lamellar twin-matrix microstructure by dislocation and γ-α’ interphase boundaries occurred at the edge (Figure 7c2). Apparently, with a reduction of up to 90%, the dislocation cell microstructure (Figure 7a3) and lamellar austenite-martensitic microstructure (Figure 7b3) were still observed in the center. However, at the edge, the globular mainly martensitic microstructure was attained after a reduction of 80–90% (Figure 7c3).

**Figure 8:**
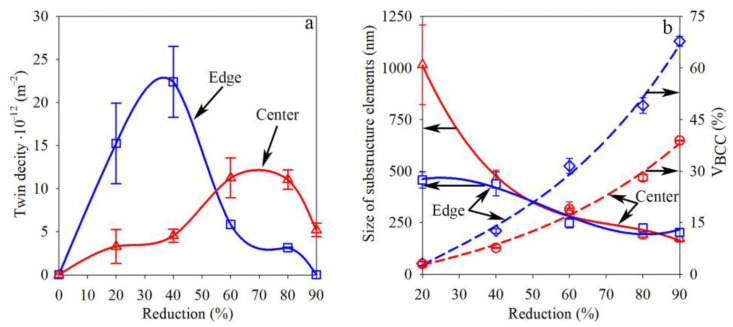
Effect of swaging on (**a**) twin density and (**b**) the average size of the substructure elements and the BCC-phase volume fraction.

It should be noted that the twin density increased more dramatically after swaging with a reduction of 20–40% at the edge in comparison to the center (Figure 8a). However, twin density at the edge decreased sufficiently with the subsequent reduction. Obviously, the maximum twin density in the center was reached just after a reduction of 60–80% followed by a slight decrease. As was also derived, with an increase in reduction to 80–90%, the average size of the substructure elements in the center and edge decreased sufficiently and tended to saturate at a value of ~200 nm, while the BCC-phase volume fraction increased gradually (Figure 8b).

## 4. Discussion

According to the received results (Section 3), cold rotary swaging resulted in the formation of a distinct phase composition gradient (Figure 3), a pronounced texture gradient of the FCC- (austenite) and BCC-phases (α’-martensite) (Figure 5), and a non-uniform hardness distribution (Figure 6) and microstructure morphology along the rod diameter (Figure 7). The observed effects were caused by the features of the processing and deformation mechanisms developed in the program steel. Dominant deformation mechanisms, and thereby plasticity, depend on the value of stacking fault energy (SFE) [29,34,35]. The SFE value of the program steel was earlier estimated at 13–20 mJ/m^2^ [36], which suggested the development of deformation-induced martensitic γ → α’ transformation and mechanical twinning. Therefore, finite element analysis (FEA) was required to discover the main features of cold rotary swaging that might provide further insights into the effect of applied processing on the microstructure and texture evolution.

### 4.1. Finite Element Analysis of Cold Rotary Swaging

**Figure 9:**
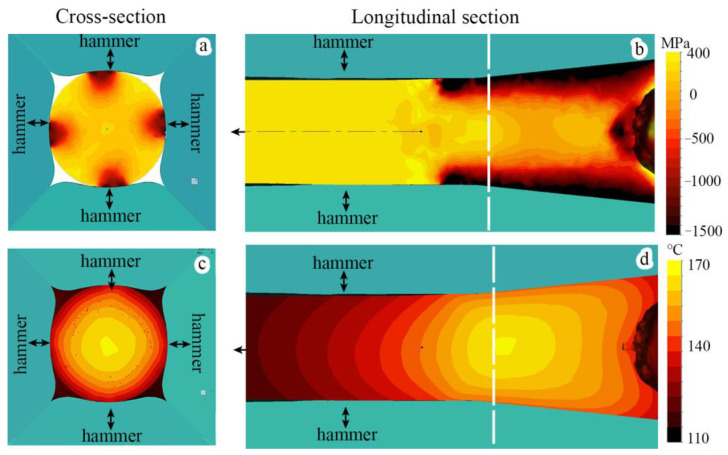
(**a**,**b**) Tensile–compressive stress distribution and (**c**,**d**) temperature during the fourth step of cold rotary swaging. White dashed lines in (**b**) and (**d**) mark the location of cross-sections ((**a**) and (**c**), respectively).

The results of the FEA are shown in Figure 9. FEA simulated that high compressive stresses were attained at the rod subsurface zone during processing (Figure 9a,b), while moderate tensile stresses were observed in the rod center. Therefore, it is expected that the main plastic strain was accumulated within the subsurface zone (Supplementary Materials Figure S3). Hence, a further increase in reduction was associated with the enhancement of the plastic strain gradient in the radial direction. It is worth noting that the quantitative estimation of plastic deformation using the applied software was limited by the experimental results from the database where true strain (e) did not exceed 2. Therefore, the modeling of deformation with e >2 might be considered as only qualitative analysis. It is worth noting that heating of the rod was also provided during swaging. According to the FEA results, the rod center might be heated up to ~200 °C (Figure 9c,d). Apparently, the temperature gradient from the edge to the center of the workpiece was attained due to outer water cooling and inner heating.

### 4.2. Phase Composition Gradient Evolution

During cold rotary swaging, the obvious gradient of phase composition was obtained after a reduction of 60% (Figure 3a) that was associated with a gradual increase in the ferromagnetic BCC-phase volume fraction from the center to the edge. However, in the as-received condition, the BCC-phase volume fraction was uniformly distributed along the diameter at a value of ~2%, which was likely associated with the presence of δ-ferrite. Subsequent cold rotary swaging activated SIMT might be developed via the γ → α’ and/or γ → ε → α’ pathways [29,37,38,39]. Obviously, SIMT occurred via both pathways. On one hand, a few points with a confidence index (CI) ~0.1 corresponding to ε-martensite were detected in the program steel after the first steps of swaging. Due to the negligible amount of ε-martensite, any reflections were not detected in the XRD patterns (Figure 3b). On the other hand, many areas of only α’-martensite in the microstructure with CI ≥ 0.1 were attained. The obtained observations were congruent with the previous results [37,40]. 

**Figure 10:**
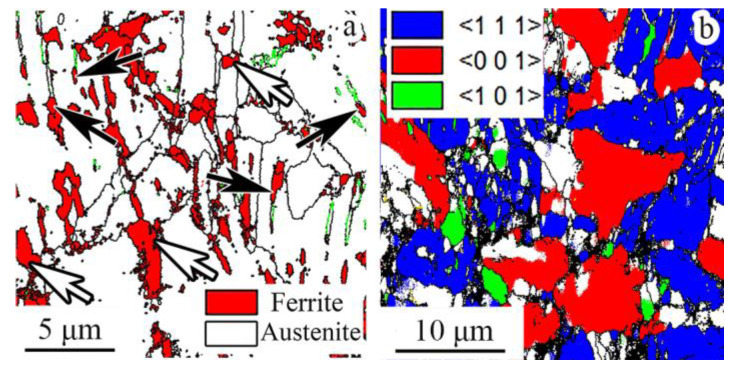
(**a**) The phase and (**b**) crystal direction maps of the rod center after a 60% reduction. High-angle boundaries (misorientation more than 15°) are marked in black and green, respectively. In (**a**), twin (Σ3) boundaries are marked in green.

The volume fraction of the strain-induced α’-martensite depended on the accumulated plastic strain [41]. It is worth noting that more plastic strain was accumulated at the rod edge in comparison to the rod core (Supplementary Materials Figure S3). Therefore, more α’-martensite was detected at the surface layers, while a gradual decrease in the α’-martensite volume fraction was observed in the center direction (Figure 3a). The nuclei of strain-induced α’-martensite were predominantly found on twins or in deformation bunds (Figure 10a), which was congruent with the results presented in [31,42]. Hence, due to higher twin density after a reduction of 20–40% (Figure 8a) and a higher current stress under blowing by hammer (Figure 9a,b), SIMT was more enhanced at the edge (Figure 3a), which caused decreasing twin density (Figure 8a). Meanwhile, SIMT within the untwined grains might also occur from the grain boundaries to the core (Figure 10a). 

Moreover, the temperature distribution can also affect SIMT and mechanical twinning. Evidently, heating increased the SFE value and therefore inhibited SIMT and mechanical twinning, providing dislocation slip [34,43,44]. Despite outer water cooling, excessive heating of the rod center during processing was predicted by the FEA (Section 4.1). Hence, the heating restricted SIMT herein, while, at the edge, SIMT might be provided more pronouncedly. Thus, with an increase in the reduction of up to 90%, the enhancement of the temperature gradient and the gradient of plastic strain accumulation promoted the α’-martensite gradient (Figure 3a). 

### 4.3. Non-Uniform Hardness Distribution

Throughout the rod cross-sections, an obvious non-uniform microhardness distribution after various swaging modes was attained (Figure 6). Increasing microhardness to the edge was found, which can be associated with more plastic strain accumulation and α’-martensite volume fraction. However, the content of α’-martensite did not increase the hardness value dramatically due to the similar hardness level of strain-induced martensite and parent matrix austenite [45]. Meanwhile, a local maximum in the center and minimum at the half radius was observed in the plots of microhardness distribution, also received after a reduction of 40–90%. The current deviations could be ascribed to sufficient residual stresses after applied processing. As previously shown [46], in the rod center, compressive residual stresses were derived, while the tensile residual stresses were attained at the edge. Interestingly, the compressive and tensile residual stresses were offset by each other at the half radius, which defined the minimum microhardness herein (Figure 6).

### 4.4. Texture Gradient Evolution

Cold rotary swaging provoked the formation of the strong axial texture of austenite with 〈111〉 and 〈001〉 components in the center after a reduction of ~60–80%, while, at the edge, only weak one component (〈111〉) texture of austenite was derived (Figure 5a,b). As predicted by the FEA (Section 4.1), different stress conditions were obtained in the center and edge during processing. Therefore, uniaxial moderate tensile and high compressive stresses were attained in the center and edge, respectively (Figure 9a). Evidently, 〈111〉-oriented austenitic grains possessed many twins inside (Figure 10b), whereas 〈001〉-oriented austenitic grains were twin-free or negligible twinned. As shown in [47], the deformation of single 〈111〉-oriented austenitic crystals promoted the twinning of austenitic stainless steel 316L, whereas austenitic crystals of the 〈001〉-orientation twinned only after a strain of 10%. According to [48,49,50], the 〈111〉 texture component of austenite could be developed by mechanical twinning under uniaxial tension, whereas the 〈001〉 component was associated with the enhancement in dislocation slip. Interestingly, a twin orientation relationship was found between the 〈001〉- and 〈111〉-grains of austenite [40]. Hence, upon tensile stress, the simultaneous development of mechanical twinning and dislocation slip within different grains induced the formation of the strong axial two-component (〈111〉 and 〈001〉) texture of austenite in the rod center. 

**Figure 11:**
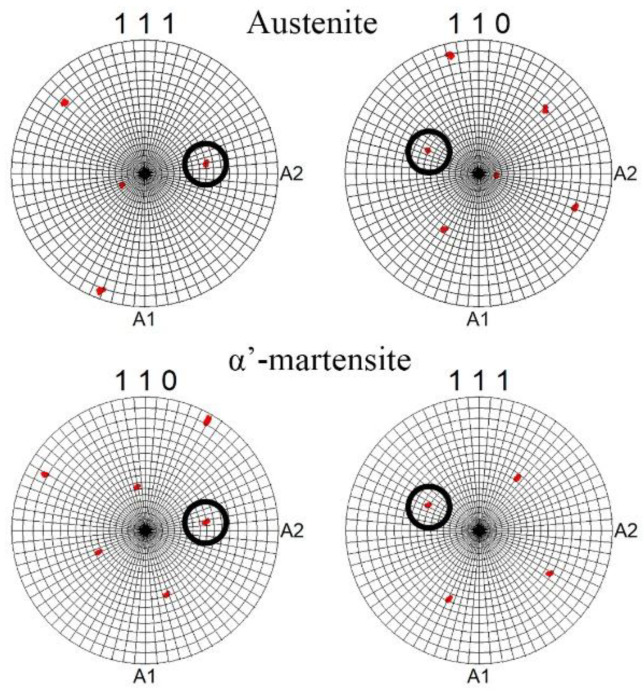
Typical pole figures of the austenite and α’-martensite crystals after a 20% reduction to show the austenite–α’-martensite relationship. The coincidences of the austenite and α’-martensite projections are indicated by the black circles.

Meanwhile, at the edge, a weaker axial 〈111〉 texture was certainly associated with the overall mechanical twinning of austenitic grains, and thereby the absence of twin-free grains. On the other hand, dislocation slip was developed herein, which might obviously reduce the effect of twinning on the enhancement of the 〈111〉 texture component of austenite. The latter was verified by KAM maps (Figure 4), where the local lattice distortions were more pronounced at the rod edge. Evidently, a decrease in the 〈111〉 texture of austenite with increasing reduction can also be ascribed to the development of SIMT. Therefore, after an 80–90% reduction, the new strong axial 〈101〉 texture of α’-martensite was enhanced throughout the rod cross-section (Figure 5c). According to the present results (Figure 11) and previous papers [2,5,37,51], austenite and α’-martensite demonstrated an orientation relationship as per Kurdjumov–Sachs (<111>α//<110>γ and {110} α//{111}γ), although there were many areas with a high confidence index (more than 0.1) of α’-martensite. Interestingly, since a few points of ε-martensite with a CI more than 0.1 were detected during EBSD analysis, a strong orientation relationship was not found between the HCP phase and FCC phase. Thus, it is expected that SIMT within the 〈111〉-oriented austenitic grains promoted the nucleation and growth of 〈101〉-oriented α’-martensitic grains (Figure 10b). Thereby, a gradual decrease in the 〈111〉 texture component of austenite was accompanied by an increase in the 〈101〉 texture component of α’-martensite (Figure 5b,c).

### 4.5. Microstructure Transformation

As shown in the present work (Section 3), the development of dislocation slip, mechanical twinning, and SIMT occurred during cold rotary swaging. Due to the various plastic strain accumulation (Supplementary Materials Figure S3) and stress conditions in the center and edge (Figure 9a), the following stages of microstructure transformation could be divided: (i) dislocation cell formation (Figure 7a1)/single system twinning (Figure 7b1) in the center and the lamellar twin-matrix microstructure (Figure 7c1) at the edge after a 20–40% reduction; and (ii) enhancement of the dislocation cell microstructure (Figure 7a3)/lamellar austenite-martensite microstructure by several system twining and SIMT (Figure 7b3) in the center and the globular, mostly martensitic microstructure (Figure 7c3), at the edge after an 80–90% reduction. Additionally, the transition condition from a lamellar to globular microstructure might be distinguished after a 60% reduction (Figure 7c2). According to Section 4.4, it is obvious that the development of a dislocation cell microstructure in the center was associated with the formation of the 〈001〉 texture component of austenite, whereas the lamellar twin-matrix microstructure of austenite promoted the enhancement of the 〈111〉 texture component of austenite.

Meanwhile, the microstructure refinement effect was also found during processing (Figure 8b). As was revealed, the average size of the substructure elements decreased with an increasing reduction. Due to the development of mechanical twinning by many systems after a 20% reduction at the edge (Figure 8a), the microstructure refinement was more pronounced herein, while mainly dislocation slip and poor twinning occurred in the rod center. As was previously found [52], the dislocation slip was obtained at the plastic deformation onset of the austenitic stainless steels with subsequent twinning development because of the lower critical shear stress compared with mechanical twinning [53]. However, a subsequent reduction resulted in the similar average size of the substructure elements throughout the cross-section that was caused by dislocation slip, twinning, and SIMT. Therefore, the progress of microstructure refinement was associated with the formation of interphase and twin boundaries as well as dislocation cells. For instance, in the 〈111〉-oriented single crystals, the saturation of mechanical twinning was followed by the formation of a dislocation cell microstructure [47] that, in the present work, occurred at the edge and resulted in the microstructure fragmentation and decreasing twin density. Moreover, SIMT caused interphase boundary formation, and therefore microstructure refinement. With an increase in the reduction to 80–90%, the average size of the substructure elements in the center and edge tended to saturate at a value of ~200 nm (Figure 8b), which was certainly the limit of microstructure refinement by the applied swaging modes. 

A further reduction in diameter might likely result in the formation of new surface and inner defects (cracks and pores) of the rod. Obviously, the development of SIMT can also occur throughout the rod cross-section. However, shear banding was expected only at the edge due to the high compressive stresses herein. Unless the rod is destroyed during the following processing, a trend to obtaining a more uniform structure along the rod diameter could be observed with increasing swaging reduction.

## 5. Conclusions

The evolution of the microstructure and texture gradient of a 321-type MASS during cold rotary swaging was explored in the current study. The following conclusions were obtained:

1. During cold rotary swaging, moderate tensile stresses were attained in the center, while high compressive stresses were predicted by FEA at the edge. Thereby, increased plastic strain accumulation at the rod edge in comparison to the rod core was expected. Due to water cooling of the rod surface and heating of the rod center during processing, a temperature gradient was also obtained.
2. Higher strain accumulation at the edge and the development of the temperature gradient during processing caused the development of the pronounced α’-martensite gradient after a 90% reduction, where the BCC-phase volume fraction varied from ~40% in the center to ~70% at the edge.
3. A strong axial two-component (〈111〉 and 〈001〉) texture of austenite was obtained in the center that turned to the weak axial one component (〈111〉) texture of austenite to the edge. Therefore, the volume fraction of the 〈111〉-oriented austenitic grains increased to a maximum of 40–50% after a 60–80% reduction with a subsequent decrease, while the volume fraction of the 〈001〉-oriented austenitic grains reached a maximum of ~32% after an 80% reduction. With an increase in reduction to 60%, the volume fraction of the 〈101〉-oriented grains of α’-martensite increased along the radial direction. Thus, the pronounced texture gradient of austenite and α’-martensite was formed after a reduction of 80–90%.
4. A non-uniform microstructure was developed during cold rotary swaging with the following stages: (i) dislocation cell formation/twinning in the single system in the center and the lamellar twin-matrix microstructure of austenite at the edge after a 20–40% reduction; and (ii) enhancement of the dislocation cell microstructure/lamellar austenite-martensite microstructure by several system twining and SIMT in the center and globular, mostly α’-martensitic microstructure, at the edge after an 80–90% reduction. With an increase in reduction to 90%, the average size of the substructure elements in the center and edge tended to saturate at a value of ~200 nm.

## Data Availability

Not applicable.

## Supplementary Materials

The following supporting information can be downloaded at: https://www.mdpi.com/article/10.3390/ma16041706/s1, Figure S1: Pole figures of austenite after cold rotary swaging with a reduction of 20%, 60%, and 90%; Figure S2: Pole figures of α’-martensite after cold rotary swaging with a reduction of 20%, 60%, and 90%; Figure S3: Distribution of accumulated plastic strain in longitudinal rod section during (a) first (20%), (b) second (40%), (c) third (60%), and (d) fourth (80%) step of swaging.
