# Supplementary material for: Gradient Microstructure and Texture Formation in a Metastable Austenitic Stainless Steel during Cold Rotary Swaging
Source: Materials (Basel). 2023 Feb 17;16(4):1706. doi: 10.3390/ma16041706 (PMC9961053; doi:10.3390/ma16041706)
Supplement: Supplementary file 1 [file materials-16-01706-s001.zip › materials-2208590-supplementary.pdf]

materials

Supplementary Materials

Gradient Microstructure and Texture Formation in a Metastable Austenitic Stainless Steel during Cold Rotary Swaging

Dmitrii Panov [1,*], Egor Kudryavtsev [1], Stanislav Naumov [1], Denis Klimenko [1], Ruslan Chernichenko [1], Vladimir Mirontsov [1], Nikita Stepanov [1], Sergey Zherebtsov [1], Gennady Salishchev [1] and Alexey Pertcev [2]

[1] Laboratory of Bulk Nanostructured Materials, Belgorod State University, 85 Pobeda St., 308015 Belgorod, Russia
[2] Department Chief Metallurgist, Perm Scientific-Research Technological Institute, 41 Geroev Khasana St., 614990 Perm, Russia
* Correspondence: dimmak-panov@mail.ru

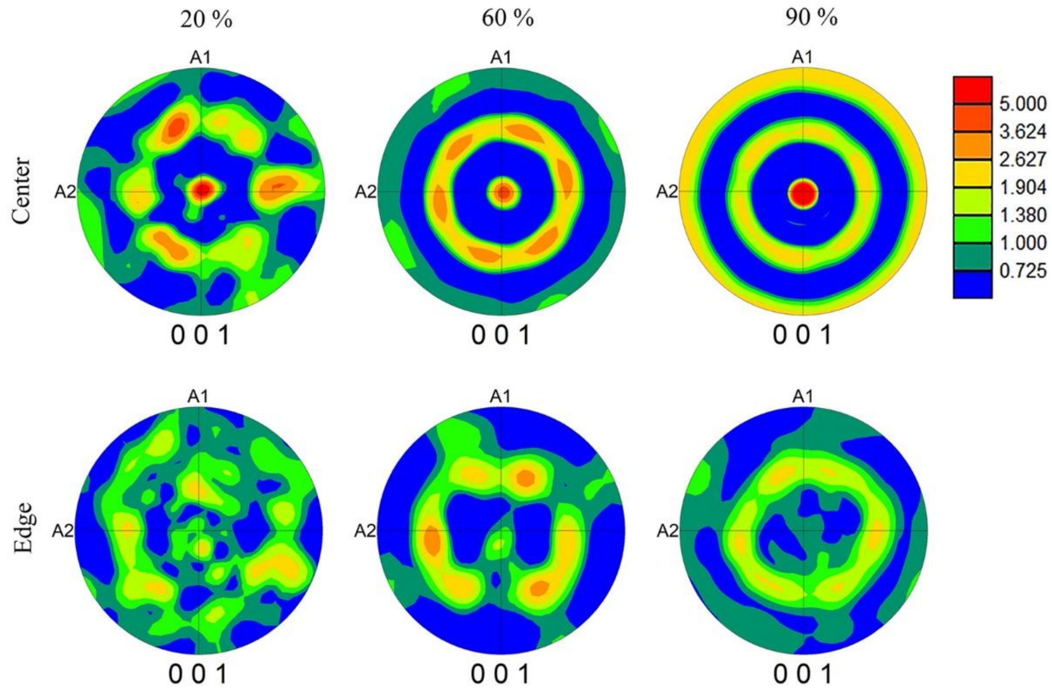

Figure S1. Pole figures of austenite after cold rotary swaging with a reduction of 20%, 60%, and 90%.

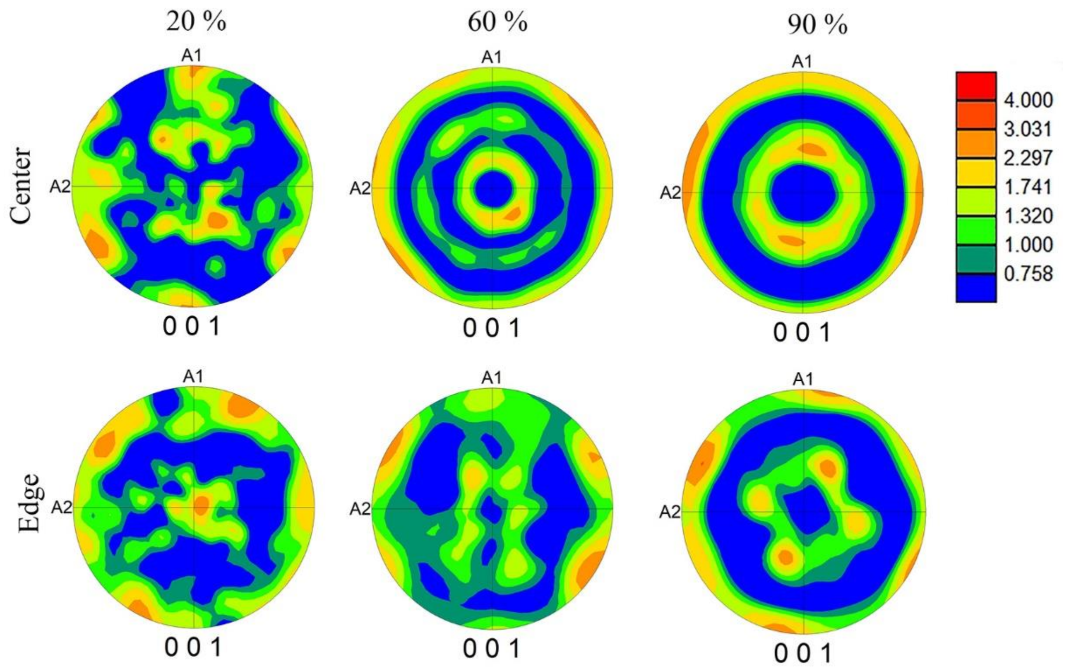

Figure S2. Pole figures of α'-martensite after cold rotary swaging with a reduction of 20%, 60%, and 90%.

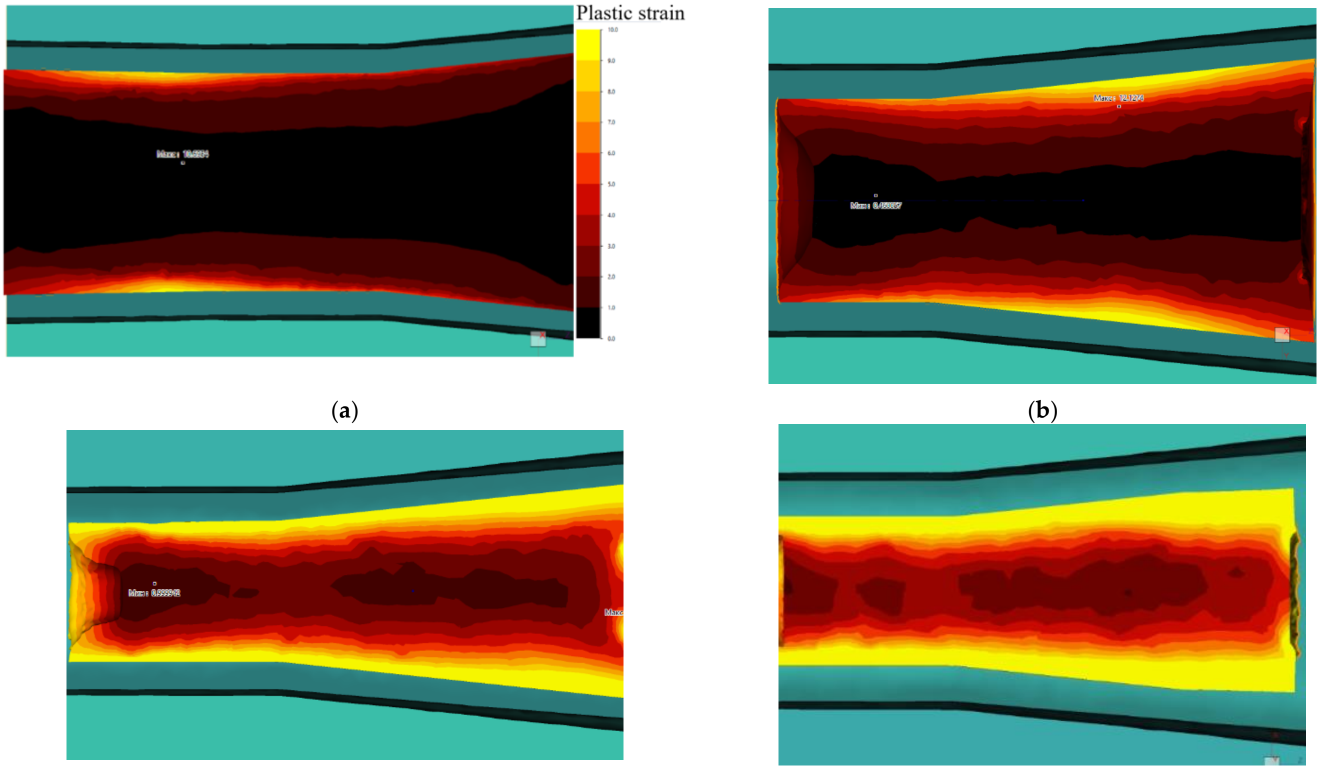

(**a**) (**b**)

(**c**) (**d**)

Figure S3. Distribution of accumulated plastic strain in longitudinal rod section during (**a**) first (20%), (**b**) second (40%), (**c**) third (60%), and (**d**) fourth (80%) step of swaging.

Disclaimer/Publisher's Note: The statements, opinions and data contained in all publications are solely those of the individual author(s) and contributor(s) and not of MDPI and/or the editor(s). MDPI and/or the editor(s) disclaim responsibility for any injury to people or property resulting from any ideas, methods, instructions or products referred to in the content.
